# Collaboration on evidence synthesis in Africa: a network study of growing research capacity

**DOI:** 10.1186/s12961-021-00774-2

**Published:** 2021-09-19

**Authors:** Jiayi Pan, Yongqi Zhong, Sarah Young, Nynke M. D. Niezink

**Affiliations:** 1 Department of Statistics and Data Science, Carnegie Mellon University, Baker Hall, Pittsburgh, 15213 PA United States of America; 2 Department of Epidemiology, University of Pittsburgh, 130 De Soto Street, Pittsburgh, 15261 PA United States of America; 3 Carnegie Mellon University Libraries, 5000 Forbes Avenue, Pittsburgh, 15213 PA United States of America; 4 Present Address: Data Science Institute, Fu Foundation School of Engineering and Applied Science, Columbia University, New York, NY United States of America; 5 Present Address: Department of Epidemiology, Bloomberg School of Public Health, Johns Hopkins University, Baltimore, MD United States of America

**Keywords:** Africa, Capacity-building, Co-authorship networks, Cross-sector collaboration, Evidence ecosystem, Evidence synthesis, Research collaboration, Social network analysis, South–South collaboration, Systematic reviews

## Abstract

**Background:**

Evidence-based practice in medicine and social policy relies heavily on evidence synthesis. To translate evidence into practical guidelines for low- and middle-income countries, local expertise is essential. The objectives of this study are to assess the change in capacity for conducting evidence synthesis in Africa and to identify key African institutions for regional capacity-building. We take on a network perspective, considering that the position of an institution in the African evidence ecosystem is one constituent of its research capacity.

**Methods:**

We systematically identified 3548 evidence synthesis publications between 2008 and 2019 with at least one author in Africa from the Web of Science Core Collection. These articles involved 3769 institutions. Longitudinal institution-level collaboration network data were constructed based on co-authorship information. We used social network analysis to examine the institutions’ connectivity and tendency for intra- and interregional collaboration. We also identified the degree- and betweenness-central African institutions and explored the structure and composition of their local network neighbourhoods.

**Results:**

The number of African institutions involved in evidence synthesis has increased substantially over the last decade, from 31 in 2008 to 521 in 2019, and so has the number of evidence synthesis publications with authors in Africa. African institutions in the evidence ecosystem have also become more connected during this period. Although the amount of intercontinental collaboration continues to exceed that of regional collaboration, the tendency for African institutions to collaborate with partners in Africa is increasing. We identified seven institutions—in South Africa, Egypt and Uganda—as central to the collaboration networks between 2008 and 2019, all of whom showed a tendency to collaborate across sectors.

**Conclusion:**

The development of more regionally based network-building initiatives would help to foster communities of practice and inter-institutional collaboration, strengthening regional research capacity. Moreover, the analysis in this study adds depth beyond a simple bibliometric analysis and illustrates that network analysis could provide a useful tool to evaluate the effectiveness of capacity-building strategies and programmes in the future.

## Background

With the dramatic increase in academic publishing rates over the past three decades, the clear synthesis of research outcomes has become increasingly important. Evidence synthesis is essential for those outside academia, as it translates results from primary studies to policy-relevant information. For example, systematic reviews can help physicians practice evidence-based medicine [1] and can help policy-makers make better decisions to reach global development targets [2]. The growing demand for evidence synthesis is reflected in the number of published studies that systematically find, assess and synthesize existing research evidence. A title search for “systematic review” in Web of Science reveals a 70-fold increase in the number of systematic reviews published annually since the year 2000 in a wide range of disciplines [3].

While this proliferation of evidence synthesis has largely been driven by researchers in North America and Europe, there is a need to increase the production of evidence synthesis by researchers and institutions in low- and middle-income countries (LMICs) [4]. Knowledge of geographic and sociocultural contexts is key for the interpretation of findings from evidence syntheses and their relevance in contexts other than those in which the research was conducted. The production of policy- or practice-relevant evidence synthesis also requires engagement with multiple stakeholders both in and outside of academia, including policy-makers, technical experts and the industrial sector. Thus, if questions relevant to LMICs are to be effectively addressed by evidence synthesis, collaborations should include researchers and other stakeholders from these regions and across sectors [5].

The Africa Evidence Network conducted a survey in 2017 to assess the growing capacity for evidence synthesis in Africa building on the work of Oliver et al. [4]. They found capacity for a wide range of evidence synthesis methods across the continent and in many sectors including academic, governmental and nongovernmental organizations (NGOs) [6]. Such surveys can be effective at identifying barriers and facilitators to conducting evidence synthesis, but can be subject to survey response biases. Thus, bibliometric approaches that comprehensively analyse publication output can provide an additional and complementary source of information about research capacity. Several studies have evaluated the production of evidence synthesis in specific research areas (see [7–9] for some recent examples), but, to our knowledge, no work has comprehensively assessed production of evidence synthesis by geographic region or with a focus on LMICs. Moreover, little is known about the structure or nature of collaborations on evidence synthesis. In this paper, we fill this gap by analysing the development of the capacity for evidence synthesis in Africa—not only considering the publication volume of institutions, but also the network of evidence synthesis collaboration.

In Africa, significant work has been done in the past decade to orient regional policy-making toward evidence-based approaches and to lift barriers to the production and use of research evidence [10–13]. For example, Cochrane Africa was established in 2017 connecting four evidence synthesis hubs in the sub-Saharan region with an aim of increasing South–South collaboration and the uptake of evidence in policy- and decision-making in healthcare [10]. Moreover, organizations like the Africa Evidence Network (regional), the Africa Centre for Systematic Reviews and Knowledge Translation (Uganda) and the Africa Centre for Evidence (South Africa) have sought to establish active communities of practice and knowledge sharing [11–13].

Connecting people is an aim many of the initiatives mentioned above share. Various global development initiatives (e.g., the Global Evidence Synthesis Initiative) have established organizational networks to improve the production and uptake of evidence synthesis and communication between decision-makers and researchers. These evidence networks have been argued to create better understanding, increased capacity, and greater potential for change, and thus to lead to better decision-making [14]. In the context of evidence synthesis, social networks play an important role, not only for the dissemination of results to policy-makers, but also for the actualization of evidence synthesis projects. Apart from providing fruitful soil for research, collaboration networks among scientists induce the spread of knowledge and skills [15]. Given the scope of evidence synthesis work, collaboration is vital, since the time, knowledge, and skills of a single researcher are limited. The need to access the knowledge and skills of others means that it is not only the quality of an individual or an institution that is relevant to the success of a project, but also the quality of the social structure in which the individual or institution is embedded, that is, his or her social capital [16, 17]. The social capital created by inter-institutional collaboration is thus an important constituent of the research capacity of institutions in LMICs.

Collaboration networks, as measured by co-authorship on scientific papers, depict a rough image of the distribution of social capital in a research field. They have been used to study the growth in research and development of research capacity in LMICs in various areas (e.g., health policy and systems research [18], research on dengue fever [19] and neglected diseases [20], and medical device innovation [21, 22]). Boshoff [23] argued that jointly coauthored papers between African and European or United States researchers can be regarded as evidence of research capacity strengthening, and that papers solely authored by African researchers are even stronger evidence of research capacity. This argument further motivates the use of a relational (or network) approach to study evidence synthesis capacity development in Africa.

In this paper, we describe the evolution of scientific collaboration on evidence synthesis in Africa between 2008 and 2019. In particular, we consider the changes over time of (1) the collaboration network connectivity, (2) the level of collaboration of African institutions within and outside Africa, as motivated by the arguments of Boshoff [23], and (3) the presence of central institutions in Africa. Although there are numerous ways to measure centrality, two types of centrality are especially relevant in this context: the extent to which an institution itself is well connected (a “hub”) and the extent to which an institution can serve as a “bridge” for knowledge between otherwise disconnected communities. Central institutions in the evidence synthesis domain may indicate opportunities for investment in training and outreach or for building capacity amongst supporting methodological experts such as librarians and statisticians. For those institutions identified as central, we investigate the nature of their collaborations, including their regional diversity and sector diversity (e.g., academic, NGO, medical). We conclude by discussing the results and possible implications for capacity-building strategies to strengthen regional and cross-sector collaboration on evidence synthesis.

## Methods

We evaluated the development of collaboration on evidence synthesis in Africa between 2008 and 2019 based on co-authorship information from published evidence syntheses that include at least one author affiliated with an African institution. Since we are interested in the development of evidence synthesis capacity of institutions over time, we chose to examine collaboration at the institutional level, rather than the author level. We used social network analysis to study the evidence synthesis collaborations, treating institutions as network nodes which are connected when they share the authorship of a paper [24, 25].

### Data collection

Data were obtained from the Web of Science Core Collection. Given the proliferation of evidence synthesis methods beyond systematic reviews in the last two decades [26], we searched for different types of evidence syntheses in the title field of article records. We included terms for what we considered to be some of the more prevalent forms of evidence synthesis, rather than an exhaustive list such as that provided in Sutton et al. [27]. We chose not to include meta-analyses as these are often carried out without a systematic literature review. We used the following search string: (“systematic review” OR “scoping review” OR “systematic map” OR “realist review” OR “evidence map” OR “evidence gap map” OR “evidence and gap map” OR “mapping review” OR “mixed methods review” OR “rapid review” OR “systematized review” OR “umbrella review” OR “evidence synthesis” OR “systematic literature review”). We limited our search to articles published between 2008 and 2019 and with at least one author based in Africa, using a search for African country names in the author affiliation field. The complete search strategy can be found in Additional file 1.

The search yielded a total of 3648 records, with 51 records removed because they had no affiliation to African countries (e.g., the name of an African country appeared in another part of the address, but the affiliation was not in Africa). The final data set contains 3597 publications and includes studies across disciplinary domains. Approximately 80% of the papers are related to health sciences and medical research (as indicated by the Web of Science research area), as evidence synthesis remains primarily a method used in these fields.

### Data preparation

For each of the extracted evidence synthesis articles, we recorded the title, authors and the authors’ affiliations for analyses in the statistical software environment R (v3.6.2) [28]. We cleaned and standardized institution information as follows. First, we matched the country names in the authors’ affiliations with the ISO-3166 standard [29] and used the ISO region information to indicate whether an institution is based in Africa. We manually reviewed and coded the unmatched country names. Second, given that some institutions appeared in the data under multiple names, we standardized the institution names using approximate string matching (e.g., see [30]) and manual checking. Approximate string matching is an approach to find text entries that include similar patterns and differ only in a small number of character insertions, deletions, or substitutions. In this way, for example, “Univ Cape Town” was matched with “Univ Cape Town Hlth Sci”. We also manually checked the potential matches for each institution. Finally, for the central institutions in the networks (as defined later in this section), we classified their sector of operation and that of their collaborators into eight categories: universities, hospitals, governments, intergovernmental organizations, research institutes, private entities (i.e., for-profit company), nonprofit organizations, and research networks (e.g., Cochrane) (see Additional file 1: Table S1 for the complete coding scheme). We treated each institution as a separate entity, regardless of whether it corresponded to a single author (i.e., authors with multiple affiliations listed) or multiple authors.

### Network construction and visualization

Based on the 2008 to 2019 co-authorship data, we created institutional-level collaboration networks using the igraph [31] package in R, and we visualized the networks using Gephi [32]. Eleven networks were constructed using a 2-year window (e.g., the edges in the 2008–2009 network result from co-authorship in 2008 and 2009). Since the average time to complete an evidence synthesis is over 1 year [33], these networks are a more realistic approximation of the collaborations among institutions than networks constructed based on a single year of publication data.

Institutions are included in all networks after their first evidence synthesis publication, as from then on we consider them part of the evidence synthesis research system. However, the 2603 institutions that published only in a single year are left out in later years, as they did not play a significant role in the research system. Leaving these institutions out does not substantially affect the results of the analyses discussed below. Forty-nine publications involving more than 20 authors were also excluded, because such collaborations likely do not reflect strong relations between institutions, and for these large collaborations, it is unclear how a publication reflects the evidence synthesis capacity of the institutions involved.

### Network analysis

After conducting a detailed, country-level analysis of the change in number of institutions involved in evidence synthesis in Africa between 2008 and 2019, and their research output in this field, we studied the evolution of the networks of collaboration on evidence synthesis.

#### Connectivity

Social network analysis provides tools to examine how closely nodes in a network are connected. A detailed description of the measures discussed in this section, as well as other measures of connectivity, can be found in [25]. To measure the extent to which the African evidence synthesis research community is integrated, we determined the proportion of institutions in the largest connected components of the 11 collaboration networks between 2008–2009 and 2018–2019, and the average path lengths within these components. A connected component of a network is a subset of the network in which there is a path between all pairs of nodes in the subset, and this subset is disconnected from any other node not in the component. The largest connected component (LCC) of a network is the connected component with the highest number of nodes. If the proportion of institutions that is in the LCC is large, this is one indication of an integrated research community.

Note that even if a network is composed of a single component, it can still show low connectivity.^1^ Therefore, we also consider the average (shortest) path length between nodes in the largest connected component. The shortest path length between two nodes is defined as the minimal number of edges needed to “walk” from one node to the other in the network. If the proportion of institutions in the LCC is large *and* the average path length within the LCC is small, this indicates that the research community is strongly integrated. Moreover, to examine the influence of non-African institutions on the connectivity of African institutions, we analysed these two measures for both the collaboration networks including and excluding the non-African institutions.

#### Intra- and intercontinental collaboration

To understand the role of intercontinental collaboration in evidence synthesis in Africa, we identified the collaborations across and within regions for the 10 networks between 2008 and 2019. We refer to collaborative ties between institutions in different regions—in this case, between African and non-African institutions—as external ties. Internal ties are collaborative ties between institutions in the same region. A classical measure of the dominance of external over internal ties (or heterophily) is the E-I index by Krackhardt and Stern [34], which is given by

1 $$\begin{aligned} \text {E-I index} = \frac{E - I}{E + I}, \end{aligned}$$

where *E* and *I* denote the total number of external and internal ties, respectively. In the context of this study, the E-I index is a measure for across-region collaboration. The possible values for this index range from $-1$ to 1. An E-I index of $-1$ indicates that all ties are internal (i.e., all collaboration happened among African or among non-African institutions), while a value of 1 implies that all ties are external. If the ties are equally divided over these groups, the index is 0.

The E-I index is a network-level index. However, since we are primarily interested in the collaboration trends of African institutions, we here propose a group-level variation on the E-I index: the *regionalization index*. The regionalization index $R_A$ of Africa is

2 $$\begin{aligned} R_A = \frac{2 \cdot I_A - E_A}{2\cdot I_A + E_A}, \end{aligned}$$

where $I_A$ denotes the number of ties within Africa, and $E_A$ is the number of ties between institutions in Africa and institutions not in Africa. Note that while the E-I index measures dominance of external over internal ties, the regionalization index does the opposite. The regionalization index can range between $-1$ and 1, with a high index indicating that collaborations occur more within Africa than between African and non-African institutions.

The regionalization index is an adaptation of the nationalization index of Binz, Truffer and Coenen [35], which does not contain the factor 2 in the numerator and denominator of (2). We propose this alteration, since the interpretation of a 0 value for the nationalization index is unexpected: for Africa to have an index of 0, the institutions in Africa need to collaborate on average with twice as many African institutions as non-African institutions.

The regionalization index has a more intuitive interpretation: it is 0 if institutions collaborate on average an equal amount with institutions within and outside Africa.^2^ This interpretation is equivalent to the interpretation of a value of 0 for the original E-I index [34]. The average proportion of within-region (internal) ties of the institutions in Africa can be expressed in terms of the regionalization index as $(1+ R_A)/2$.

#### Central institutions

To identify institutions that play an important role in the collaboration networks, we calculated the degree and betweenness centrality for all institutions. The degree centrality of a node in a network is defined as the number of edges of that node. Institutions with a high degree centrality—that is, with a large number of collaborative ties—are referred to as “hubs”. The betweenness centrality of a node *v* is defined as

3 $$\begin{aligned} C_B(v) = \sum _{\begin{array}{c} s,t : \\ s\ne t \ne v \end{array}}\frac{\sigma _{st}(v)}{\sigma _{st}}, \end{aligned}$$

where $\sigma _{st}$ denotes the number of shortest paths between two nodes *s* and *t*, and $\sigma _{st}(v)$ denotes the number of shortest paths between the two nodes that pass through node *v* [36]. The summation in (3) runs over all distinct pairs of nodes that are different from *v*. The betweenness centrality of a node indicates to what extent it serves as a “bridge” between other nodes.

Although betweenness and degree centrality scores are often correlated (nodes with many ties have more chances to serve as a bridge), nodes with high betweenness centrality do not necessarily have high degree centrality. For example, when an institution links to one institution in each of two otherwise disconnected clusters, its degree centrality is only two, but its betweenness centrality is very high.

Degree centrality and betweenness centrality are naturally dependent on network size (i.e., the number of nodes in a network). Given that nodes in larger networks have more opportunities for connections, the centrality scores of central nodes in large networks are likely to be larger than those of central nodes in smaller networks. Since the collaboration networks we consider here grow in size over time, we also calculated the normalized degree and betweenness centrality scores for each institution, which divide the (unnormalized) centrality score by the maximum centrality score possible for a node in a network with the same number of nodes.^3^

After computing the degree and betweenness centrality scores for all institutions, we identified all African institutions that ranked top three among the African institutions in either of the centrality measures in any of the collaboration networks between 2008–2009 and 2018–2019. We refer to these institutions as the (most) central institutions and analysed how their centrality scores, as well as their numbers of publications, changed over time.

Finally, we constructed the local network neighbourhoods (also known as “ego networks”) of the most central African institutions based on the 2018–2019 network. The local neighbourhood of an institution is composed of the collaborators of that institution, together with the ties among the collaborators. To understand the kinds of other institutions the central institutions collaborate with, we examined the region and sector (e.g., university, hospital, nonprofit) of the institutions in their local network neighbourhoods.

## Results

Between 2008 and 2019, a total of 3597 evidence synthesis publications appeared, on which at least one individual from an African institution collaborated. These publications involve 4181 institutions from 154 countries. The number of institutions involved per paper ranged between 1 and 103. We excluded the 49 publications (1.4%) with more than 20 authors from further analysis (see Additional file 1: Figure S1 for the distribution of authors per paper). The remaining 3548 publications involve 3769 institutions from 145 countries.

**Fig. 1:**
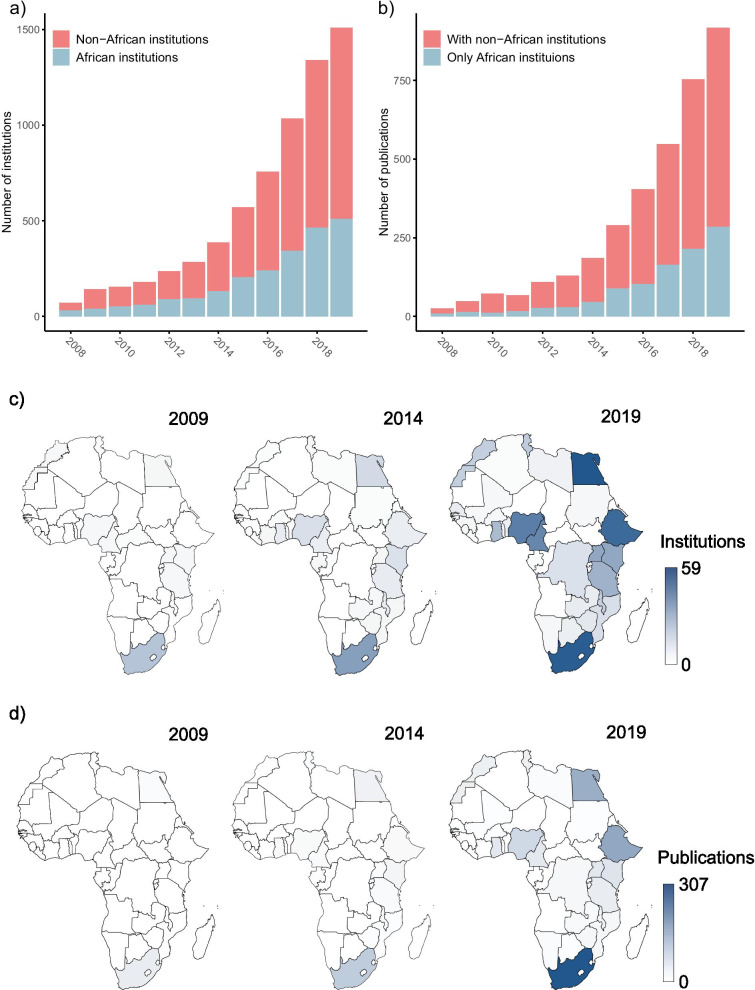
Evolution of evidence synthesis research in Africa between 2008 and 2019. **a** Number of African and non-African institutions. **b** Number of publications authored by solely African institutions and jointly by African and non-African institutions. **c** Number of institutions publishing evidence syntheses by country. **d** Number of evidence synthesis publications by country

The number of institutions producing evidence syntheses increased from 71 in 2008 to 1508 in 2019, with an increase from 31 to 509 for African institutions specifically (see Fig. 1a). The number of evidence synthesis publications increased from 26 in 2008 to 917 in 2019, with a greater increase in publications jointly authored by African and non-African institutions compared to publications authored solely by African institutions (Fig. 1b). The number of African countries producing evidence syntheses increased over time, but not homogeneously over the continent, as shown in the maps in Fig. 1c, d. In Africa, the five countries showing the greatest increase in the number of institutions publishing evidence synthesis research between 2008 and 2019 are Egypt (56 more institutions), Ethiopia (53), Nigeria (44), Cameroon (41), and South Africa (36). These are also among the countries with the largest increase in evidence synthesis output, with South Africa, Ethiopia, and Egypt showing the greatest increase in output. Notably, while Egypt and South Africa had about the same number of institutions producing evidence syntheses in 2019 (59 versus 57), the South African institutions published 307 papers in 2019, twice as many as the Egyptian institutions (149 papers).

### Network analysis

**Fig. 2:**
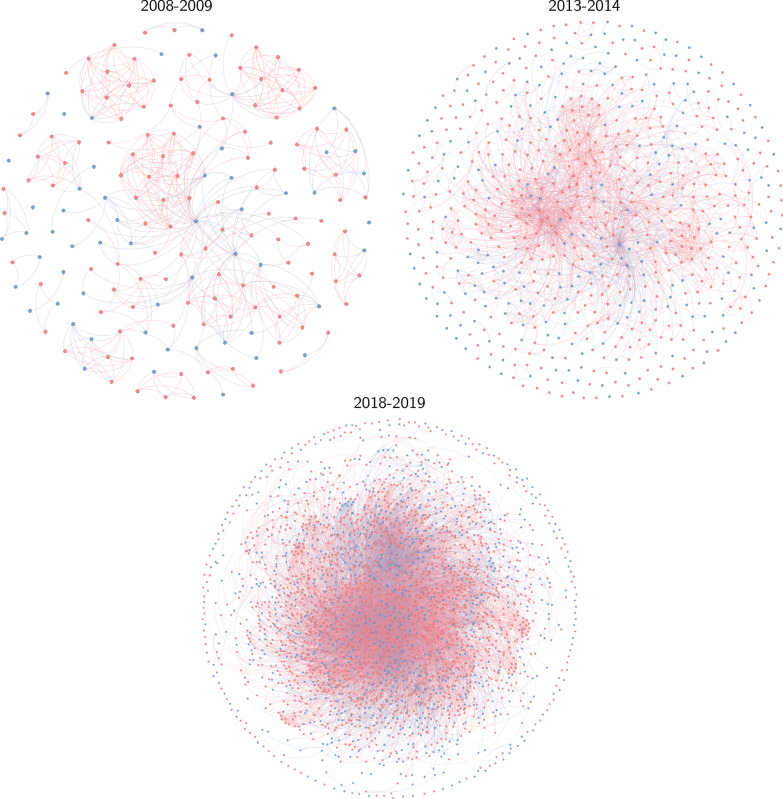
Networks of collaboration on evidence synthesis for 2008–2009, 2013–2014, and 2018–2019. Blue nodes denote African institutions, and pink nodes denote non-African institutions

The collaboration networks as derived from joint publications in 2008–2009, 2013–2014 and 2018–2019 are shown in Fig. 2. The total number of collaborative ties increased from 530 in the 2008–2009 network to 13331 in the 2018–2019 network. On average, the number of institutions per publication increased from 3.6, with a standard deviation (SD) of 2.7 in 2008–2009 to 4.1 (SD=2.8) in 2018–2019, indicating that not only is there more collaboration on evidence synthesis in general, but there are also on average more parties involved in the publication of individual papers.

**Fig. 3:**
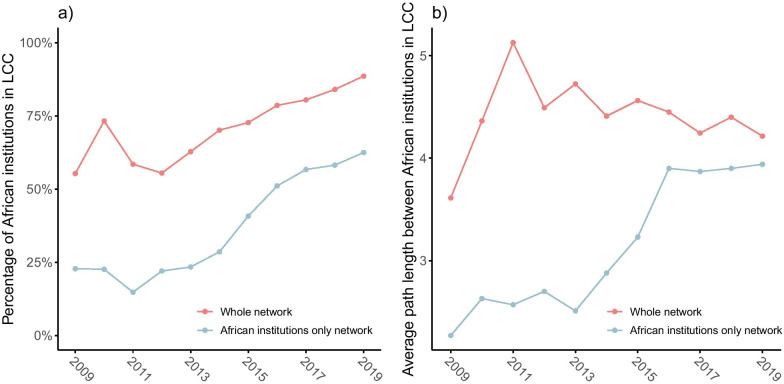
Between 2008–2009 and 2018–2019, **a** the percentage of African institutions in the largest connected component (LCC) of both the whole networks and the network with only African institutions increased, and **b** the average path length in the LCC of the whole network decreased, while it increased in the network with only African institutions

#### Connectivity

As shown in Fig. 3a, the percentage of African institutions in the largest connected component (LCC) of the collaboration network with all institutions increased from 45.6% in 2008–2009 to 86.5% in 2018–2019. In the collaboration network composed of only African institutions, the percentage of African institutions in the LCC increased from 22.8 to 62.5%. These increasing trends suggest the gradual integration of the evidence synthesis research community. However, the lower percentages in the networks with only African institutions indicate that some African institutions are connected only via non-African institutions.

This finding is supported by Fig. 3b, which shows the average path length between African institutions in the LCCs of the collaboration networks with all institutions and with only African institutions. Despite the increase in the size of the LCC, the average path length within the LCC in the network including all institutions shows an overall decreasing trend after 2011. This indicates that the evidence ecosystem is becoming more integrated. However, the average path length of the LCC in the collaboration network with only the African institutions increased, almost up to the average path length of the whole network. Given that the LCC of the complete network contained about 25% more of the African institutions than the LCC of the network with only African institutions in 2018–2019, the difference in average path length of only 0.4 extra tie indicates that the non-African institutions play an important role in keeping the African community closely connected.

#### Intra- and intercontinental collaboration

**Fig. 4:**
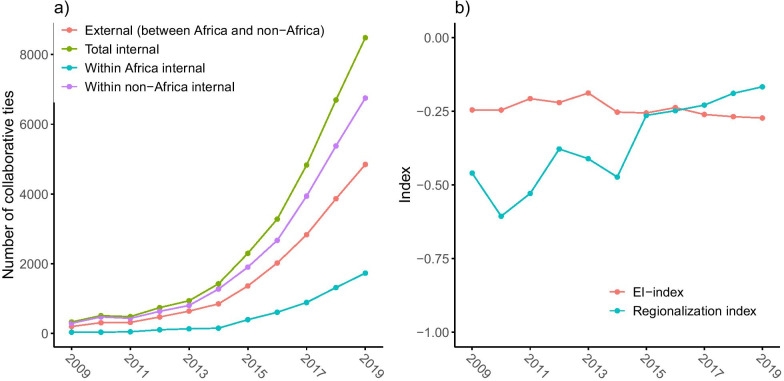
Intra- and intercontinental collaboration. **a** Number of various types of collaborative ties. **b** Indices of integration of the African and non-African research community. Higher values of the E-I index indicate a dominance of collaboration between African and non-African institutions over that among African institutions and among non-African institutions. Higher values of the regionalization index indicate the tendency of African institutions to collaborate more within than outside Africa

Figure 4a shows the number of (external) collaborative ties between African institutions and non-African institutions, and also the number of internal collaborative ties within the African community and the non-African community. We find that for African institutions, the number of ties with non-African institutions is consistently more than twice the number of ties with other African institutions. Consequently, the regionalization index for Africa is strictly negative (see Fig. 4b). That is, African institutions tend to have on average more collaborative relations with non-African institutions than with other African institutions. However, the increasing trend of the regionalization index indicates a weakening of this tendency. The regionalization index increased from $-0.46$ in 2008–2009 to $-0.17$ in 2018–2019. Correspondingly, the average proportion of within-region (internal) ties of the institutions in Africa was 0.27 in 2008–2009, but increased to 0.42 in 2018–2019.

Figure 4b also depicts the evolution of the E-I index. The negative value of the E-I index, which does not change much over the years, suggests a regional homophily in collaboration.^4^ This surprising finding can be explained by the large amount of (internal) collaborations among the non-African institutions. These outnumber both the collaborations between non-African and African institutions and the (internal) collaborations among African institutions.

#### Central institutions

Table 1 shows the evolution of the rank in degree and betweenness centrality for the most central African institutions (with non-African institutions excluded from the ranking). We observe three patterns in the rank evolution. First, University of Cape Town, University of the Witwatersrand and Stellenbosch University from South Africa consistently remained high in rank in both degree centrality and betweenness centrality. Among the three, University of Cape Town is the most stable with its degree centrality always ranking first and its betweenness centrality ranking first or second. Second, the two universities from Egypt, Cairo University and Assiut University, both experienced a drop in their degree and betweenness centrality between around 2011 and 2015, and later showed some recovery. Note that the drop in the rank of Assiut University was much larger than that of Cairo University, and its recovery was much weaker. Third, Makerere University from Uganda and University of KwaZulu-Natal from South Africa both ranked high for several years, but then declined in rankings.

If we were to include the non-African institutions in the ranking based on centrality scores, several non-African institutions would be frequently identified as central in the collaboration networks (see Additional file 1: Table S2). For example, the London School of Hygiene & Tropical Medicine ranked consistently higher than the African central institutions. WHO—the only intergovernmental institution among the central institutions—developed its central status in 2012–2013, but its ranking began to drop from 2017–2018 on. The central position of the non-African institutions in the collaboration networks is remarkable, given the fact that all publications included in this study have at least one author from an African institution. This further illustrates the importance and influence of non-African institutions in the African evidence ecosystem.

**Fig. 5:**
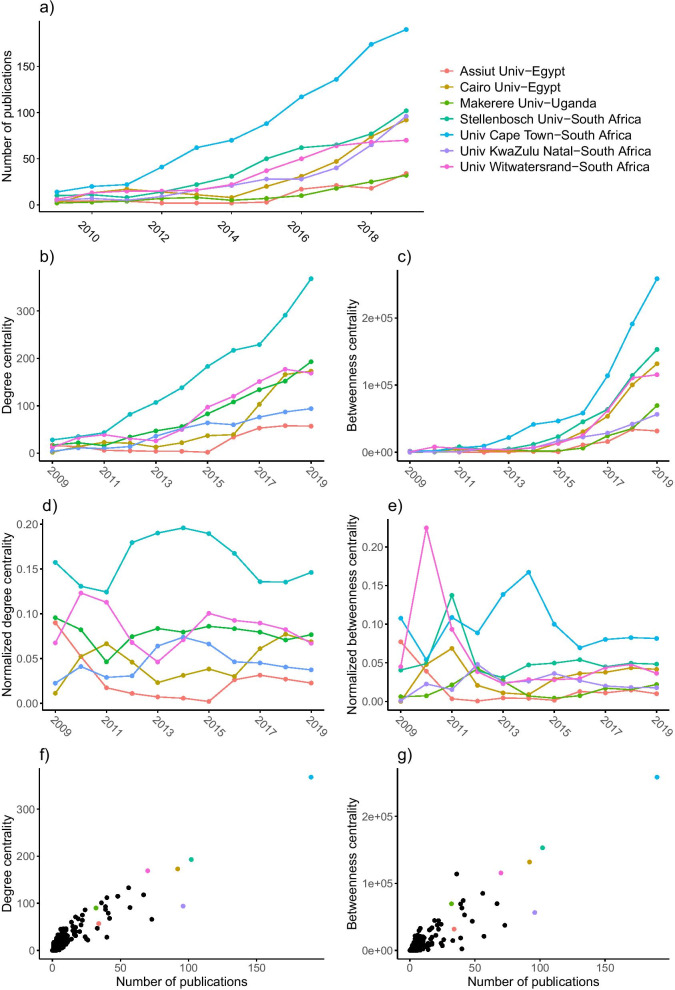
For the most central African institutions, changes in **a** the number of publications; **b** degree centrality; **c** betweenness centrality; **d** normalized degree centrality; **e** normalized betweenness centrality from 2008–2009 to 2018–2019 for central institutions. **f** Association between degree centrality and the number of publications. **g** Association between betweenness centrality and the number of publications in 2018–2019 with central institutions denoted in colour

While so far we have regarded the ranking of the central institutions, Fig. 5 shows the actual values of the degree and betweenness centrality scores for the most central African institutions. Figure 5b shows that the number of institutions with whom the African central institutions collaborate increases over time. The same is true for the betweenness centrality of these institutions (see Fig. 5c). The normalized centrality scores, however, accounting for the growth in the number of institutions in the research system, stabilize over time (see Fig. 5d, f, e).^5^ The stabilizing normalized degree centrality indicates that the growth rate in the number of collaborative ties of the hubs is equal to the growth rate in the number of institutions. The stabilizing normalized betweenness centrality indicates that there are no collaborations established elsewhere in the network that make the central universities lose their bridge position.

**Table 1:** Changes in rank of degree centrality (top) and betweenness centrality (bottom) for institutions that have ranked top 3 at least once in terms of the centrality measures respectively

Degree rank	2009	2010	2011	2012	2013	2014	2015	2016	2017	2018	2019
UCT-South Africa	1	1	1	1	1	1	1	1	1	1	1
SU-South Africa	2	3	4	2	2	2	3	3	3	4	2
UW-South Africa	5	2	2	3	5	4	2	2	2	2	4
CU-Egypt	36	4	3	6	14	7	7	8	4	3	3
AU-Egypt	3	4	14	35	54	77	194	13	11	16	24
UKN-South Africa	21	9	7	7	3	3	4	6	7	9	10

Betweenness rank	2009	2010	2011	2012	2013	2014	2015	2016	2017	2018	2019
UCT-South Africa	1	2	2	1	1	1	1	1	1	1	1
SU-South Africa	4	3	1	4	2	2	2	2	2	2	2
UW-South Africa	3	1	3	5	5	3	6	4	3	3	4
CU-Egypt	21	4	4	7	7	13	5	3	4	4	3
AU-Egypt	2	5	14	25	16	18	41	12	15	11	23
UKN-South Africa	15	6	7	2	4	4	3	5	8	8	12
MU-Uganda	11	11	6	3	3	15	27	21	9	10	9

*UCT* University of Cape Town, *SU* Stellenbosch University, *UW* University of the Witwatersrand, *CU* Cairo University, *AU* Assiut University, *UKN* University of KwaZulu-Natal, *MU* Makerere University

Note that the centrality history of University of Cape Town departs from that of the other central universities. The university established a notable position in 2012, due to a comparatively large increase in number of collaborations with other institutions, but did not gain many ties in 2016–2017, resulting in a drop in normalized degree centrality. Its relatively large increase in number of collaborations in the first few years also causes University of Cape Town to have a notable peak in normalized betweenness centrality in 2014–2015. However, after 2014, it loses its bridge position somewhat due to connections being made elsewhere in the research system. For example, the more central positions of non-African institutions, such as Oxford University and University College London, increase in betweenness centrality in these years, and thus take on more of a bridge position (see Additional file 1: Table S2).

All African central institutions, and especially University of Cape Town, show considerable growth in research output, as indicated in Fig. 5a. However, the number of publications and the centrality measures are not a one-to-one relationship: high productivity does not necessarily indicate high centrality. For example, University of KwaZulu-Natal published more papers than University of the Witwatersrand in 2018–2019, yet it is less degree and betweenness central (Fig. 5f, g). Inconsistencies like this reflect the advantage of network analysis in identifying institutions that potentially play important roles in the African evidence ecosystem and yet would have been missed by bibliometric analysis.

**Fig. 6:**
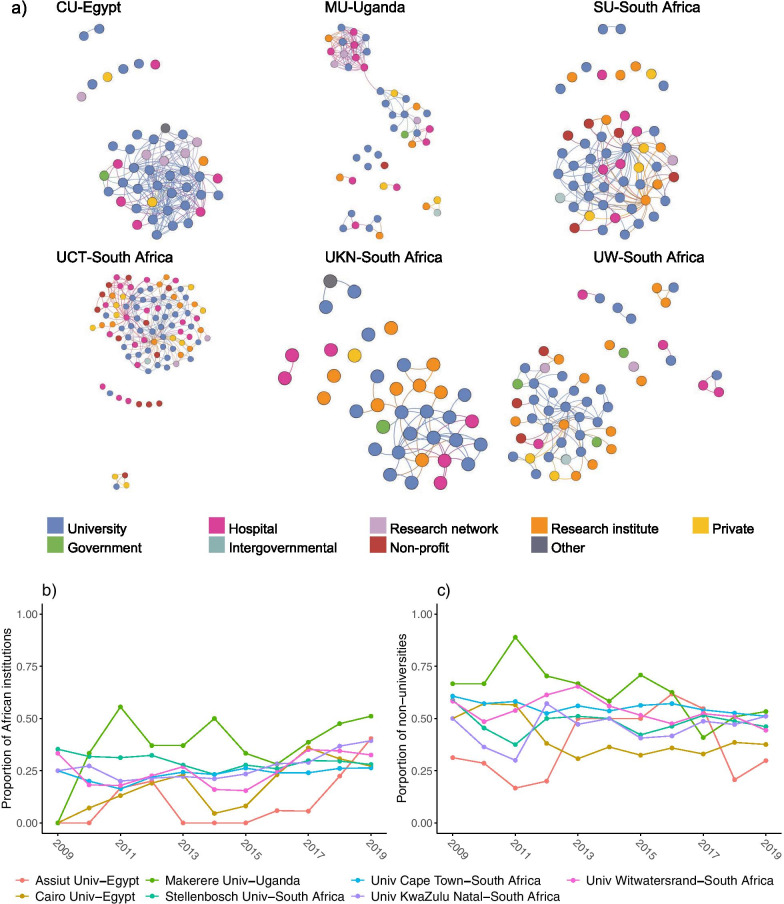
**a** Local network neighbourhoods (only the African collaborators) for the African central institutions. **b** Changes in the proportion of African institutions and **c** changes in the proportion of nonacademic institutions in the local network neighbourhoods

The local network neighbourhoods of the African central institutions based on collaboration in 2018–2019 are shown in Fig. 6. To visualize the extent to which the central institutions connect other African institutions, we exclude the non-African collaborators from these graphs. Note that, although the local neighbourhoods of the central institutions are shown separately, some of these networks do overlap. The local network of Assiut University is not shown as it lost its central role in the evidence synthesis research system after 2010–2011.

The local network neighbourhoods of most of the African central institutions are composed of a densely connected largest component that contains the largest proportion of the institutions in the local network, and some isolated institutions and very small components. Makerere University from Uganda (Figure 6a) is the exception here: the largest component in its local network is composed of two clusters, and its second largest component contains six institutions. Makerere University thus plays a key role in connecting otherwise disconnected communities. Furthermore, considering that Makerere University has the largest proportion of African institutions in its local network neighbourhood among all central institutions since 2009–2010 (Fig. 6b), its importance in bridging collaboration between African institutions is evident. For no other African central institution did the percentage of regional collaborators ever exceed 50%.

For all the African central institutions, universities make up the largest part of their local network neighbourhood (Fig. 6c). Notably, from 2008–2009 to 2015–2016, the local network neighbourhood of Makerere University is more than 50% composed of nonacademic institutions—the highest percentage among all African central institutions. However, after 2015–2016, this number decreases, and universities start making up a larger part of its local network neighbourhood.

## Discussion

Strengthening the capacity of LMICs to produce evidence syntheses has become a priority to tackle the social, environmental and health challenges of these regions more effectively. We used social network analysis to assess collaboration on evidence synthesis publications in Africa and found increasing collaboration on evidence synthesis within Africa, with a considerable and sustained role for non-African institutions. Certain African institutions and countries are well-situated to serve as evidence synthesis hubs based on evidence synthesis production trends and the quantity and diversity of collaborations.

Our analysis indicates that the increasing trend in the global production of evidence synthesis [3] is also reflected in African research over the past decade. Since 2008, the number of African institutions and countries with researchers involved in producing evidence synthesis has grown dramatically. This may reflect the increased emphasis on evidence-based practice and policy-making globally, but also regional efforts to increase capacity for evidence synthesis through training and network-building [37].

South Africa and Egypt represent the majority of evidence synthesis publications, which is unsurprising given their higher gross domestic product (GDP) [38] and greater research and development (R&D) investment compared to the rest of the region [39]. However, while in both countries a large number of institutions are involved in evidence synthesis, South Africa produces by far the most evidence synthesis publications in Africa. In South Africa, significant value is placed on evidence-based decision-making and evidence synthesis, both in government and academia, as noted by Stewart et al. [40]. They indicate a demand for evidence across sectors with investment, from both internal and external funders, in fostering a vibrant “evidence ecosystem”. This is reflected in the prominence of South African institutions in the evidence synthesis co-authorship network, including the consistently central position of South African universities, as discussed further below.

We used network analysis to delve deeper into the structure and characteristics of evidence synthesis collaboration in Africa and between African and non-African institutions. We examined the connectivity of the network over time as an indication of the flow of knowledge, expertise and resources between institutions. In addition to growing numbers of collaboration on evidence synthesis, we found increasing integration of the evidence synthesis research community, suggesting greater capacity for knowledge diffusion and resource sharing across institutions. The 2008–2009 network in Fig. 2 shows numerous disconnected components, each dominated by non-African institutions. Over time, the network evolves to be much more connected, with 84% of the institutions in the network connected either directly or indirectly by 2019.

To further assess the role of collaboration with non-African institutions in evidence synthesis, we used the E-I index [34] and proposed the regionalization index to measure changes in collaborations internal and external to the region over time. We find that African institutions continue to collaborate more with non-African institutions than with other African institutions. Institutions in the Global North have remained important in evidence synthesis publishing in Africa over the last 10 years, and institutions in Europe and North America dominate the network despite the fact that our data set was built solely on publications with an Africa-based author. This result is consistent with previous findings from studies of African research. For example, in a study of research collaboration in southern Africa, Boshoff [41] noted that only 3% of papers published from 2005 to 2008 were coauthored by researchers from more than one country in the region, and of those, 60% included at least one author from a high-income country.

We used network analysis to highlight institutions that hold central network positions based on both production and collaboration measures. Central institutions contribute to the stability of the network, help connect organizations on the periphery and serve as conduits of knowledge, resources and expertise [19]. Thus, in the context of an evidence synthesis co-authorship network, central institutions may be well-primed to serve as centres for increased investment and capacity-building with the aim of strengthening regional evidence ecosystems.

The consistently central position of the South African universities in this network reflects a combination of significant investment in research compared to other countries in the region [39], and a national research agenda that emphasizes evidence, reflected in established centres and networks dedicated to evidence-based practice and evidence synthesis production [40]. For example, Cochrane South Africa has long been the only Cochrane coordinating centre in the sub-Saharan African region [10]. Stellenbosch University is host to the Centre for Evidence-based Health Care and is part of the Cochrane Africa network. The University of the Witwatersrand and the University of KwaZulu-Natal both provide systematic review training opportunities and have library services supporting evidence synthesis [42, 43]. University of Cape Town is arguably the leading research university in Africa in terms of research output and global rankings [44]. These findings agree with those of Stewart et al. [6], which found a central role for South African institutions based on a survey of evidence synthesis capacity in Africa.

Cairo University and Assiut University in Egypt also held central roles in the network. However, likely due to the economic impacts of the Egyptian Revolution in 2011 [45], their production and central positions declined. Cairo University has seen some degree of recovery in this network, but Assiut University has since produced relatively low numbers of evidence synthesis publications. Egypt also invests heavily in research and higher education, compared to other countries in the region [39], but it is unclear to what extent evidence synthesis has been emphasized at a systems level.

Makerere University in Uganda appears with a high betweenness central ranking, particularly from 2011 to 2013, indicating that it acted as a bridge between otherwise disconnected institutions. It has been found that having larger collaboration networks and collaborating with otherwise disconnected peers is correlated with more novel and highly cited research output [46]. At an institutional level, organizations that foster communication across disciplinary boundaries and extramural collaboration are associated with research productivity and scientific progress [47]. Thus, high-betweenness central institutions are also well-situated to serve as priorities for investment and potential targets for centres of excellence schemes [48]. In fact, Makerere University, at the peak of its betweenness-central ranking in 2013, established the Africa Centre for Systematic Reviews and Knowledge Translation [49]. This centre has contributed to building capacity for evidence synthesis across disciplines through trainings, priority-setting and regional network-building. This role is also reflected in the composition of their local network, as discussed further below.

For central institutions in our network, we examined the diversity of sectors represented in their local collaboration networks based on collaborations in 2018–2019, as well as the degree to which they are collaborating with regional partners (Fig. 6). Cairo University shows a mostly connected network consistently made up largely of other academic institutions and hospitals. On the other hand, Makarere University and the South African institutions show greater cross-sector diversity in their collaborations. For example, University of Cape Town shows considerable collaborations with the private and nonprofit sectors, and research institutes are common across the networks of the South African institutions. Such diverse collaborations could indicate evidence syntheses closely tied to policy development, as opposed to studies of a more clinical nature authored by teams of academic medical researchers.

### Limitations and future research

Our study relied solely on data from Web of Science. While this database provides thorough multidisciplinary coverage, it may not fully reflect the landscape of African publications, particularly those published in regional journals not indexed in mainstream databases or evidence syntheses not published in the formal peer-reviewed literature. Other databases could be searched to include broader coverage, but many insufficiently capture affiliation data for such an analysis. We also did not do an exhaustive search for all known evidence synthesis methods, but rather relied on our knowledge of the evidence synthesis publishing landscape to search for the most common forms of this type of research. We believe that the addition of other, less common evidence synthesis types would not have had much impact on the results, but this could be explored in future work.

In addition, we assumed that the publication of a systematic review or other form of evidence synthesis is a reflection of the capacity of all authors for conducting evidence synthesis. While the contribution of individual authors is not known, and the quality or impact of publications was not considered, we believe our analysis should reflect overall trends at an institutional level and thus can serve as a proxy for institutional capacity.

We also recognize that the current work lacks authorship-level contribution from Africa-based researchers. Thus, the results were interpreted based on the authors’ knowledge of co-authorship network analysis, and experience in collaborating on evidence synthesis with international teams and working with colleagues at African institutions to develop and conduct evidence synthesis training programmes. However, the paper lacks the expertise of those deeply familiar with the African research context. While this does not invalidate the results, it may have impacted the depth of interpretation.

While we focused on institutions with high degree centrality as potential targets for capacity-building, those engaging in evidence synthesis on the periphery of the network could provide an interesting focus for future research and for investment in capacity-building programmes. Another avenue for future research is the disciplinary differences in evidence synthesis collaboration. Evidence synthesis has increased across disciplines in recent years [50], and capacity may vary based on institutional strengths in certain domains. An examination of geographical, linguistic or topical groupings could explain some collaboration patterns.

In addition, research on the motivations and incentives for collaboration on evidence synthesis, and associated barriers, could provide greater insight for capacity-building strategies. Funders are known to drive the makeup of collaborations, for example, in requirements for interdisciplinary teams or the inclusion of top-tier research institutions [51, 52]. Generally, understanding why researchers in African institutions collaborate on evidence synthesis with researchers in the Global North, or with other institutions in their region and across sectors, could improve strategies for increasing South–South collaboration and contribute to the development of vibrant, regional evidence ecosystems.

### Implications for policy and practice

Our study indicates that substantial opportunity exists for leveraging existing knowledge of evidence synthesis methods in African institutions and improving regional capacity. Efforts to facilitate local and regional collaborations, train domain experts and methods specialists, and increase sustained access to funding and resources should be key elements of capacity-building programmes.

In the context of evidence synthesis, the incentives to publish and collaborate with researchers in the Global North likely continue to outweigh those to collaborate with researchers in other LMICs. Access to research databases and the journal content necessary to carry out comprehensive reviews in addition to funding, publishing opportunities, and high-profile research partnerships are all factors that likely influence decisions to collaborate on evidence synthesis. Despite the dominance of non-African collaborations in our network, collaborations within Africa are also increasing. We note potential for intra-African collaboration on evidence synthesis and an opportunity to build capacity from within, for evidence synthesis relevant to the African context. Initiatives that increase incentives and opportunities for regional collaboration, as well as enable access to necessary resources such as journal content and funding, would serve to strengthen the regional network and build capacity for regionally relevant evidence synthesis.

We found little evidence of collaboration with government agencies in these networks. Initiatives to develop more partnerships between universities and government agencies could help inform more policy-relevant evidence synthesis. That said, stakeholder engagement may not be adequately reflected in authorship, and more research is needed to understand the role of government actors in evidence synthesis production. Moreover, further analysis would be needed to determine what is driving these differences in local collaboration networks.

Oliver et al. [4] carried out an assessment of evidence synthesis production capacity in LMICs. They note a range of approaches to improving evidence synthesis capacity in these regions including training programmes, the establishment of networks of systematic reviewers and organizations, and building knowledge management systems for research sharing. At the same time, there exist challenges and barriers to capacity-building for evidence synthesis, including a lack of trained support specialists like statisticians and information specialists, language barriers and a lack of access to journal content [4]. International organizations like WHO, Cochrane and the Campbell Collaboration, as well as global networks like the Global Evidence Synthesis Initiative Network, can play a key role in lifting such barriers through training, network-building and improving access to resources. In our network, for example, WHO was prominent and held a relatively stable central position over time. Given the results of this study, we think the development of more regionally based network-building initiatives would help to foster communities of practice and inter-institutional collaboration, strengthening regional research capacity.

## Conclusions

In this study, we used social network analysis to assess the changes in the network of co-authorship on evidence synthesis publications between 2008 and 2019 to shed light on the capacity of African institutions to conduct evidence syntheses and the distribution of social capital within this network. We found the collaboration network to be increasingly connected, with greater involvement of African institutions over time and increasing collaboration amongst African institutions. The network perspective on research capacity in this study revealed key roles played by institutions like Makarere University in Uganda and the University of the Witwatersrand in South Africa in bridging otherwise disconnected institutions and establishing diverse collaborations—findings a simple bibliometric analysis of publication output would have missed. This analysis also complements survey methods, which can elucidate barriers and facilitators to research capacity and highlight the role of stakeholders not reflected in publication authorship. Given the value of regional and cross-sectoral collaboration in building vibrant evidence ecosystems, network analysis could provide a useful tool to evaluate the effectiveness of capacity-building strategies and programmes in the future, as illustrated by this study.

## Supplementary Information

**Additional file 1.** Additional figure and tables.

**Additional file 2.** Dataset.

## Data Availability

The data analysed in this study are included as supplementary file with this article (Additional file 2).

## Abbreviations

LMICs: Low- and middle-income countries
LCC: Largest connected component
WHO: World Health Organization

## Footnotes

1 Think of a “line network” in which the first node is connected only to the second, and the second only to the first and the third, etc.

2 Let $I_i$ and $E_i$ denote, respectively, the number of African and non-African collaborators of institution *i*, and suppose the total number of African institutions is *n*. The regionalization index is 0 if $2\cdot I_A = E_A$, or equivalently, $\sum _{i=1}^n I_i = \sum _{i=1}^n E_i$. Dividing both sides by *n*, we can interpret this as institutions collaborating on average an equal amount with institutions within and outside Africa.

3 For an undirected network of size *n*, these maxima are $n-1$ for degree centrality and $(n-1)(n-2)/2$ for betweenness centrality.

4 Recall that a higher value of the E-I index corresponds to more external (interregional) collaboration, while a higher value of the regionalization index corresponds to more internal (within Africa) collaborations.

5 The normalized centrality scores in the earlier years fluctuate, because of the small size of the collaboration networks in these years. A new institution or a small network change could therefore result in large changes in normalized centrality measures.
