# Supplementary material for: Collaboration on evidence synthesis in Africa: a network study of growing research capacity
Source: Health Res Policy Syst. 2021 Sep 19;19:126. doi: 10.1186/s12961-021-00774-2 (PMC8451124; doi:10.1186/s12961-021-00774-2)
Supplement: Supplementary file 1 — Additional file 1. Additional figure and tables. [file 12961_2021_774_MOESM1_ESM.pdf]

[**Additional File 1**]: Collaboration on evidence synthesis in Africa: a network study of growing research capacity

J Pan, Y Zhong, S Young, NMD Niezink

July 16, 2021

Contents

1 Search Strategy **2**

2 Supplementary Figure 1: Distribution of authors per paper **3**

3 Supplementary Table 1: Definitions of institution types **4**

4 Supplementary Table 2: Changes in rank of degree centrality (top) and betweenness centrality (bottom) for institutions that have ranked top 3 at least once in terms of the centrality measures respectively (non-African institutions included in ranking). **5**

1 Search Strategy

- Data source: Web of Science Core Collection
- Date of search: April 13, 2020
- TI = ("systematic review" OR "scoping review" OR "systematic map" OR "realist review" OR "evidence map" OR "evidence gap map" OR "evidence and gap map" OR "mapping review" OR "mixed methods review" OR "rapid review" OR "systematized review" OR "umbrella review" OR "evidence synthesis" OR "systematic literature review")

  AND

  AD = (Angola OR Benin OR Botswana OR "Burkina Faso" OR Burundi OR Cameroon OR "Cape Verde" OR "Cabo Verde" OR "Central African Republic" OR Chad OR Tchad OR Comoros OR Congo OR "Cote d'Ivoire" OR "Ivory Coast" OR Djibouti OR "Equatorial Guinea" OR Eritrea OR Ethiopia OR Gabon OR Gambia OR Ghana OR Guinea OR Kenya OR Lesotho OR Liberia OR Madagascar OR Malawi OR Mali OR Mauritania OR Mauritius OR Mozambique OR Namibia OR Niger OR Nigeria OR Réunion OR Rwanda OR "Sao Tome and Principe" OR Senegal OR Seychelles OR "Sierra Leone" OR Somalia OR "South Africa" OR Sudan OR Swaziland OR Swasiland OR Eswatini OR Tanzania OR Togo OR Uganda OR "Western Sahara" OR Zaire OR Zambia OR Zimbabwe OR Rhodesia OR Tunisia OR Algeria OR Libya OR Egypt OR "Western Sahara" OR Morocco)
- Limit: 2008-2019
- Export Fields:
  - Author(s)/Editor(s)
  - Abstract
  - Addresses
  - PubMed ID
  - Title
  - Times Cited
  - Language
  - Source
  - Document Type
  - Keywords
  - Source Abbrev.
  - Web of Science Categories
  - Conference Information
  - Research Areas

2 Supplementary Figure 1: Distribution of authors per paper

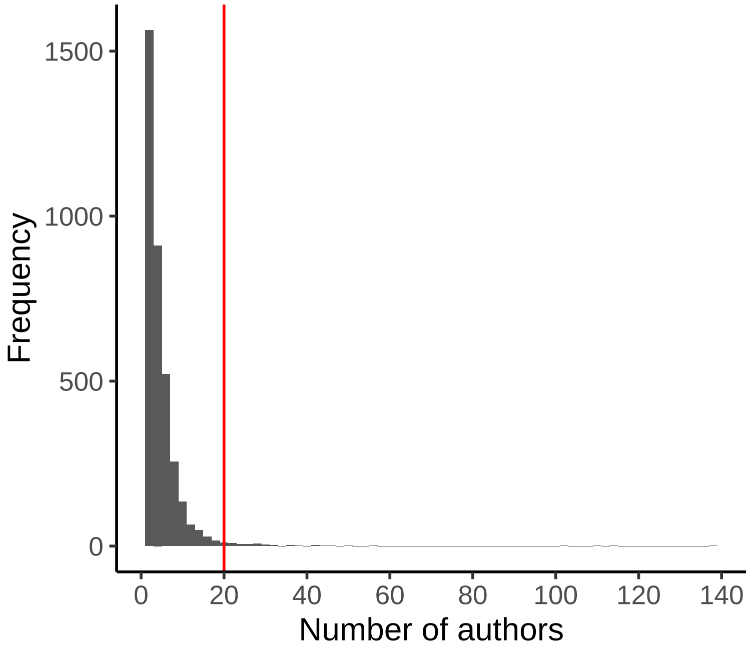

For the stability of the analysis and because mass-authored papers may be a poor indicator of institutional capacity, we removed in total 49 publications with more than 20 authors. This resulted in the removal of a total of 854 institutions from 106 countries (on average 17 institutions per publication). Of these removed institutions, 442 (52%) appear in the publications that are included in our analysis, and 152 (18%) are African institutions from 33 African countries. Of the removed African institutions, 60 (52%) were only involved in the publications with mass-authorship in the past decade.

3 Supplementary Table 1: Definitions of institution types

Institution Type	Definition	Examples
University	An academic institution, including universities or colleges	University of Cape Town; London School of Hygiene and Tropical Medicine
Hospital	Any institution providing medical care including hospitals, teaching hospitals, systems of clnics, etc.	Helen Joseph Hospital–South Africa, Child Centre Maulbronn–Germany
Research Network	Professional societies, networks and informal research organizations	Cochrane, British Association of Dermatologists
Research Institute	Formal research centers–can include government-funded research centers (e.g., NIH), those affiliated with academic institutions or non-governmental research institutes	South African Medical Research Council, Center for Disease Control and Prevention
Government	Government departments and programs, excluding government-funded research institutes	Ghana Ministry of Health, Romanian National TB Program
Private	Private sector entities (e.g., companies, LLCs), excluding private hospitals, private academic institutions and private research institutes	Evidera, GlaxoSmithKline
Intergovernmental	International organizations made up of members representing sovereign states	UNESCO, WHO
Non-profit	Organizations formed to address particular social cause, health condition, or other advocacy work without the aim of profit-making	Médecins Sans Frontières, Mothers2Mothers-South Afrca

4 Supplementary Table 2: Changes in rank of degree centrality (top) and betweenness centrality (bottom) for institutions that have ranked top 3 at least once in terms of the centrality measures respectively (non-African institutions included in ranking).

Degree rank	2009	2010	2011	2012	2013	2014	2015	2016	2017	2018	2019
UCT-South Africa	1	1	1	1	1	1	1	1	1	1	1
LSHTM-United Kingdom	2	3	3	2	2	2	2	2	5	2	3
UCL-United Kingdom	3	7	10	67	12	8	5	5	7	9	10
WHO-Switzerland	5	10	90	10	3	3	4	3	3	6	9
UW-South Africa	9	2	2	5	12	6	3	4	4	3	7
HU-United States	43	15	44	175	92	14	13	22	22	10	2
MUV-Austria	NA	109	8	3	16	441	462	554	121	173	2315
OU-United Kingdom	NA	NA	250	150	92	14	7	7	2	4	4

Betweennness rank	2009	2010	2011	2012	2013	2014	2015	2016	2017	2018	2019
UCT-South Africa	1	10	2	1	1	1	1	3	1	1	1
LSHTM-United Kingdom	2	2	3	4	2	2	2	1	4	5	2
UCL-United Kingdom	3	8	8	13	22	20	5	7	7	13	11
UW-South Africa	7	1	4	6	10	7	12	11	6	4	8
UL-United Kingdom	8	24	22	48	3	4	42	41	18	7	13
SU-South Africa	9	11	1	5	6	3	4	4	5	3	5
WHO-Switzerland	11	7	128	12	4	5	8	2	2	7	24
MU-Uganda	21	36	14	3	7	50	77	56	20	24	19
HU-United States	28	18	26	148	181	17	19	19	19	11	3
UKN-South Africa	41	21	17	2	9	8	6	12	16	18	23
KCL-United Kingdom	57	3	43	148	13	54	24	59	91	19	15
OU-United Kingdom	NA	NA	128	39	65	16	3	6	3	2	7

[1] *UCT:* University of Cape Town, *LSHTM:* London School of Hygine & Tropical Medicine, *UCL:* University College London, *WHO:* World Health Organization, *UW:* University Witwatersrand, *HU:* Harvard University, *MUV:* Medical University of Vienna, *OU:* Oxford University, *UL* University of Liverpool, *SU:* Stellenbosch University, *MU:* Makerere University, *UKN:* University of KwaZulu Natal, *KCL:* Kings college of London.
